# Systemic inflammatory response index is associated with increased all-cause and cardiovascular mortality in obstructive sleep apnea: evidence from NHANES and validation with an external hospital-based dataset

**DOI:** 10.3389/fmed.2026.1823845

**Published:** 2026-05-01

**Authors:** Mengxian Jiang, Jing Li, Xiang Gu, Wei Chen, Yexing Xu

**Affiliations:** 1 Department of Otorhinolaryngology Head and Neck Surgery, The Central Hospital of Wuhan, Tongji Medical College, Huazhong University of Science and Technology, Wuhan, Hubei, China; 2 Department of Otorhinolaryngology Head and Neck Surgery, Maternal and Child Health of Hubei Province, Tongji Medical College, Huazhong University of Science and Technology, Wuhan, Hubei, China

**Keywords:** cohort study, mortality, NHANES, obstructive sleep apnea, systemic inflammatory response index

## Abstract

**Objective:**

Obstructive sleep apnea (OSA) is increasingly recognized for its association with significant morbidity and mortality. Despite advances in understanding OSA’s impacts, the role of systemic inflammation in this context remains underexplored. The systemic inflammatory response index (SIRI) has emerged as a potential marker for evaluating inflammatory status and predicting adverse health outcomes. We aim to examine the association between the SIRI and the risks of all-cause and cardiovascular mortality in individuals exhibiting symptoms of obstructive sleep apnea (OSA), with further validation in patients with diagnosed OSA.

**Methods:**

A cohort of 9,992 adults with OSA symptoms was derived from the National Health and Nutrition Examination Survey (NHANES) for model development, and an independent external validation cohort of 994 patients with OSA was obtained from the Central Hospital of Wuhan. Multivariate weighted Cox regression, subgroup analyses, restricted cubic spline (RCS) analyses, and receiver operating characteristic (ROC) curves were employed to assess mortality risk in the exploratory cohort, with external validation performed using the Wuhan cohort.

**Results:**

In the NHANES dataset, elevated SIRI levels were identified as significant independent risk factors for all-cause mortality (adjusted HR = 1.51, 95% CI: 1.16–1.97, *p* < 0.001) and cardiovascular mortality (adjusted HR = 2.03, 95% CI: 1.17–3.54, *p* < 0.001) among individuals with OSA symptoms. RCS analysis revealed a non-linear relationship between SIRI and both all-cause and cardiovascular mortality (both *p* for non-linearity <0.001). Moreover, SIRI demonstrated substantial predictive value for all-cause mortality (AUC = 0.822) and cardiovascular mortality (AUC = 0.806) in this population. These findings were further confirmed in the external validation cohort from the Central Hospital of Wuhan. In this independent dataset, RCS analysis consistently demonstrated a non-linear positive association between SIRI and mortality outcomes, and ROC analysis yielded AUC values of 0.780 for all-cause mortality and 0.866 for cardiovascular mortality, respectively.

**Conclusion:**

SIRI serves as a significant predictor of mortality in individuals with OSA, highlighting its potential utility in early risk screening for long-term adverse outcomes, both at the population level and clinical settings.

## 1 Introduction

Obstructive sleep apnea (OSA) is a highly concerning sleep disorder, and its prevalence has shown an increasing trend year by year (1). A study on the global prevalence of OSA indicates that nearly 1 billion people worldwide are affected by this disease, and the prevalence in some countries exceeds 50%, causing a huge social and economic burden (2). OSA has been clearly proven to be an independent risk factor for cardiovascular and cerebrovascular diseases, metabolic syndrome, and (3–6). Studies have shown that the all-cause mortality of patients with untreated severe OSA significantly increases, and their risk of cardiovascular-related death can increase by 2–3 times (3, 7).

The systemic inflammatory response index (SIRI) is a novel comprehensive index reflecting systemic inflammation, which is calculated by neutrophil count multiplied by monocyte count divided by lymphocyte count (8, 9). SIRI can help assess the prognosis of tumor patients (10, 11) and patients with various non-tumor diseases including diabetes (12) and chronic kidney disease (CKD) (13). In the field of respiratory diseases, SIRI may serve as a predictive tool to assess the mortality rate of asthma patients (14). However, the role of SIRI in assessing the mortality risk of individuals with OSA symptoms remains unclear, especially from the perspective of a large sample. Notably, insights regarding the association between systemic inflammatory markers and mortality outcome from patients with clinically diagnosed OSA remain underexplored.

To address this critical gap, our study aims to investigate the association between the systemic inflammatory marker SIRI and both all-cause and cardiovascular mortality in individuals with OSA symptoms, utilizing nationally representative cohort data. To further validate the robustness and generalizability of our findings in clinically diagnosed OSA patients, we also incorporated an independent external validation cohort comprising 994 adults with diagnosed OSA from the Central Hospital of Wuhan. This research seeks to clarify the prognostic significance of SIRI within the context of OSA, ultimately providing novel insights for early intervention strategies and enhancing clinical management of affected individuals at both the public health and clinical settings.

## 2 Materials and methods

### 2.1 Data and study participants from NHANES

The current observational study followed the guidelines of Strengthening the Reporting of Observational Studies in Epidemiology (STROBE), and the completed checklist has been provided as Supplementary material. The data used in this study were obtained from the National Health and Nutrition Examination Survey (NHANES). The NHANES database is an accessible resource administered by the guidance of the National Center for Health Statistics (NCHS), which is responsible for data collection and quality control. The NHANES database uses standardized stratified and multi-stage sampling methods for data collection. The survey received formal approval from the NCHS Research Ethics Review Committee, and all participants provided informed consent before being included in the survey. Among all survey cycles of the NHANES, only the 2005–2008 and 2015–2018 cycles incorporated a comprehensive assessment of OSA-related symptoms, encompassing detailed inquiries about sleep-disordered breathing frequency, daytime somnolence, and self-reported apnea events. The remaining NHANES cycles utilize abridged sleep assessment protocols (e.g., shortened questionnaires with fewer OSA-specific items), which may compromise the validity of OSA identification, so these cycles were excluded from this study.

**Figure 1:**
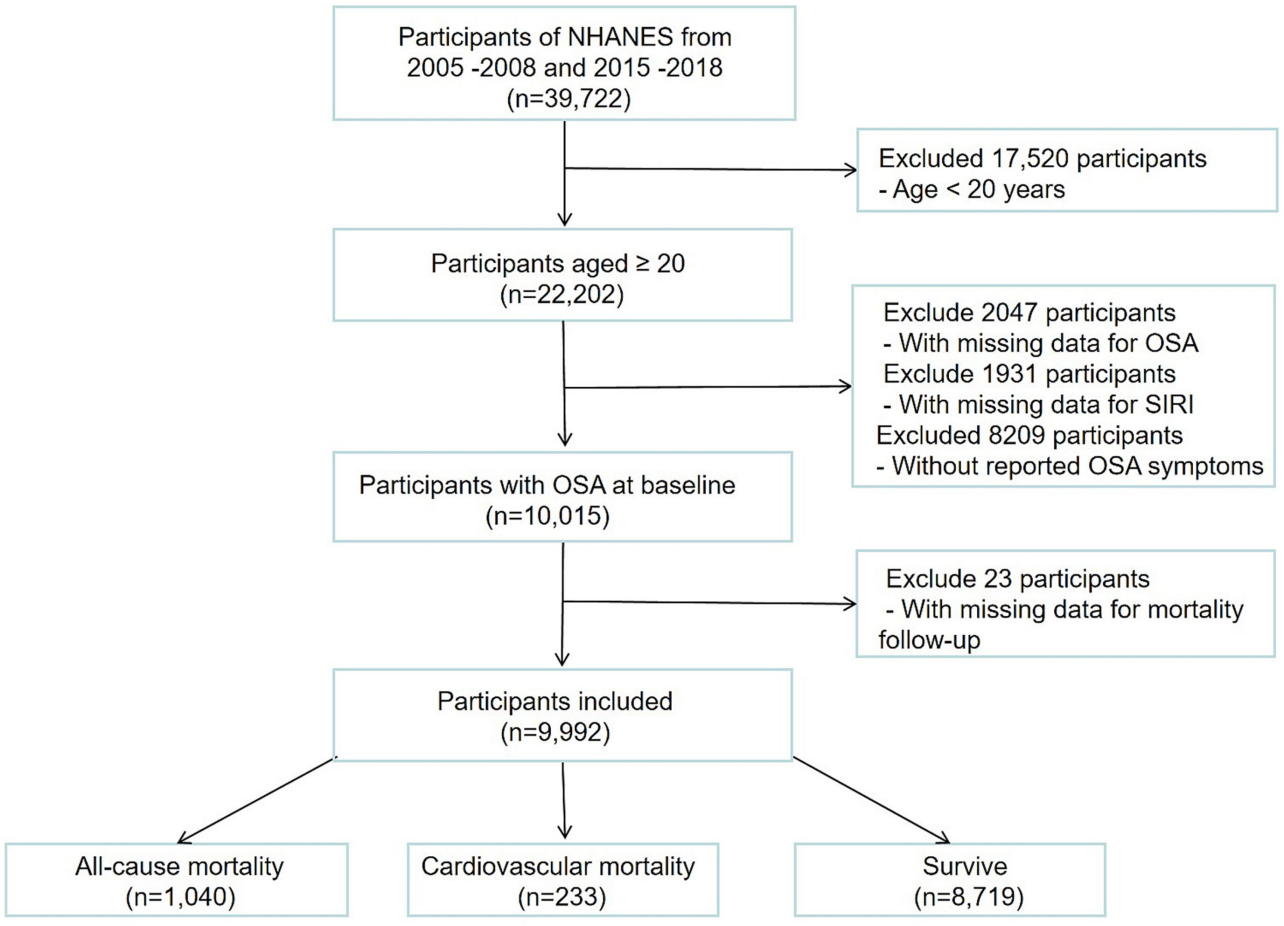
Flowchart of the study population.

Our study used data from the aforementioned NHANES waves, which included a total of 39,722 participants. We included adults aged 20 years or above with OSA. Our exclusion criteria were as follows: initially, participants aged <20 years (*n* = 17,520) were excluded. Subsequently, 2,049 participants were excluded from the analysis due to missing OSA data, and 1,931 participants were excluded due to insufficient data on complete blood count (CBC), while 8,209 participants without OSA were also excluded. Finally, 23 participants with missing follow-up data were excluded. Ultimately, 9,992 participants were included in this study (see Figure 1).

### 2.2 Ascertainment of OSA in NHANES

According to prior NHANES based studies (15–17), OSA symptoms was identified when an individual answered “yes” to at least one of the following three NHANES questionnaire items: (1) Excessive daytime sleepiness, even though the individual sleeps at least 7 h per night, with reports of 16–30 occurrences per month; (2) Reporting 3 or more episodes of gasping, snorting, or cessation of breathing per week; (3) Reporting snoring more than 3 times per week.

### 2.3 Exposure variable

The counts of neutrophils, lymphocytes, and monocytes were measured by trained personnel using an automated hematology analyzer. SIRI was calculated using the following formula (18, 19):


SIRI=(Monocyte count×Neutrophil count)/Lymphocyte count

### 2.4 Covariates

Based on previous studies (20), the covariates included multiple variables, such as age (divided into 20–39 years old, 40–59 years old, ≥60 years old), sex, race, educational level, family income and poverty ratio (PIR) (low income <1.3, middle income 1.3–3.5, high income >3.5) (21), smoking and drinking habits, body mass index (BMI) (normal weight <25, overweight 25–30, obese >30) (22), and other clinical variables. Additionally, a history of coronary heart disease, stroke, total cholesterol level, and estimated glomerular filtration rate (eGFR) were all measured in accordance with standardized protocols. eGFR was calculated using the CKD-EPI formula (23).

### 2.5 Mortality

The mortality status of the participants was verified by cross-referencing with the National Death Index (NDI, available at: https://ftp.cdc.gov/pub/Health_Statistics/NCHS/datalinkage/linked_mortality/). The life status of the participants was verified through cross-referencing with the National Death Index (NDI). The all-cause mortality rate (24) was determined based on the NDI records as of December 31, 2019, with the vital status data linked to the NHANES dataset. Cause-specific mortality rates were determined using ICD-10 codes. Cardiovascular deaths were defined as ICD-10 codes I00–I09, I11, I13, and I20–I51. Follow-up began when the participants completed the examination at the survey center, and the follow-up outcome was whether a mortality event occurred and the cause of death if it occurred. All-cause and cardiovascular mortality were selected as the primary outcomes given their established relevance in OSA populations and the strong pathophysiological link between OSA and cardiovascular disease. Other cause-specific outcomes, such as cancer-related mortality, were not included as they were beyond the scope of this study.

### 2.6 External hospital data collection

A retrospective analysis was conducted on patients aged ≥18 years who were diagnosed with obstructive sleep apnea (OSA) at the Central Hospital of Wuhan between January 31, 2019, and January 31, 2021. A total of 994 patients were included, all of whom were diagnosed with OSA based on overnight polysomnography (PSG), defined by an apnea-hypopnea index (AHI) of ≥5 events per hour. The primary inclusion criterion was adult patients (aged ≥18 years) with a confirmed diagnosis of OSA via PSG. All included participants had complete clinical data, including age, sex, ethnicity, smoking history, alcohol consumption history, body mass index (BMI), comorbidities (diabetes mellitus, hypertension, coronary heart disease), and laboratory parameters (neutrophil count, lymphocyte count, monocyte count, white blood cell count, uric acid, triglycerides, cholesterol). Clinical data were retrieved from the hospital’s electronic medical record system, and long-term prognostic outcomes and causes of death were ascertained through telephone follow-up. Patients were followed up until December 2025, with a median follow-up duration of 4.43 years. In the external validation cohort from the Central Hospital of Wuhan, the same set of covariates was assessed, including age, sex, educational level (<high school graduate, high school or above), ethnicity (Han, minority), economic status (poor, ordinary, rich) (25), marital status, smoking and drinking status, BMI (normal weight <25, overweight 25–30, obese >30) (22), hypertension, diabetes, stroke, coronary heart disease, eGFR, and total cholesterol, with descriptive characteristics summarized in accordance with standardized clinical data collection protocols. This study was approved by the Ethics Committee of The Central Hospital of Wuhan (Approval ID: WHZXKYL2025-197).

Exclusion criteria were as follows: patients with central or peripheral sleep apnea syndrome (*n* = 5), those with acute or chronic infections (*n* = 4), individuals with autoimmune diseases (*n* = 2), patients with malignant tumors (*n* = 1), and those with hematological disorders (*n* = 2). Additionally, participants with incomplete polysomnography data at the time of data collection (*n* = 8) and those receiving anti-inflammatory or immunosuppressive therapy (*n* = 4) were excluded, as illustrated in Supplementary Figure 1.

### 2.7 Statistical analysis

Analyses involving the NHANES dataset accounted for the complex, multi-stage, stratified sampling design by incorporating the appropriate sampling weights, primary sampling units (PSUs), and strata, in accordance with NCHS guidelines. Continuous variables were described with median and interquartile range (IQR) due to deviation from the normal distribution and compared with Wilcoxon test among participants with different levels of SIRI. Categorical variables were reported with frequency and percentages, with chi-square test used for comparison.

Additionally, we used the weighted Cox proportional hazards regression model to explore the relationship between SIRI and all-cause mortality and cardiovascular mortality in the OSA population. SIRI was included in the model as a numerical variable and an interquartile variable. Three models were constructed. Model A did not adjust for covariates; Model B adjusted for age, race and sex; Model C further adjusted for education level, marital status, PIR, BMI, drinking, smoking, history of coronary heart disease, stroke history, cholesterol level, eGFR, hypertension, diabetes, and coronary heart disease. Subgroup analyses were performed to evaluate the consistency of the association between SIRI and mortality across different demographic and clinical subgroups. Interaction *p*-values were derived by including multiplicative interaction terms between SIRI and each subgroup variable in the Cox regression models, with significance assessed using the Wald test. Restricted cubic spline (RCS) analyses were performed using three knots positioned at the 10th, 50th, and 90th percentiles of the SIRI distribution to evaluate the dose–response relationship between SIRI and all-cause mortality and cardiovascular mortality. The receiver operating characteristic (ROC) curve was used to explore the predictive value of SIRI for all-cause mortality and cardiovascular mortality in the OSA population. To further contextualize the predictive performance of SIRI, we compared its discriminative ability with that of high-sensitivity C-reactive protein (hs-CRP), a well-established systemic inflammatory marker, using data from the available NHANES cycles, with statistical significance assessed by DeLong’s test for correlated ROC curves; since high adiposity directly affects inflammation, BMI was also compared with SIRI. The primary findings were subsequently validated in the external cohort using the aforementioned statistical methods without considering for sample weights. In the NHANES dataset, missing covariate data (all <5% missing) were addressed using multiple imputation by chained equations (MICE) with 5 imputed datasets. In the external validation cohort, complete-case analysis was conducted due to the low proportion of missing data (<3%). Multicollinearity was assessed in the two datasets separately using variance inflation factors (VIFs), with all covariates showing VIF values below 2.5, indicating no significant multicollinearity in the adjusted models (Supplementary Tables 1, 2). All statistical analyses were performed using R statistical software (version 4.5.1; R Foundation for Statistical Computing, Vienna, Austria) with the “survival” package for Cox regression and the “rms” package for RCS analysis, and a two-sided *p*-value <0.05 was considered statistically significant.

## 3 Results

### 3.1 Population characteristics in NHANES dataset

This study ultimately included 9,992 individuals with OSA from NHANES. Among them, 5,374 were male (53.8%), with a median age of 52 years (IQR, 38–64 years); 3,522 participants (35.2%) aged ≥60 years old. During the median follow-up of 4.75 years (IQR, 2.75–12.3), there were 1,040 deaths due to all causes (all-cause mortality rate, 10.4%), among which 233 were due to cardiovascular diseases (cardiovascular mortality rate, 2.3%). The SIRI levels were divided into quartiles: Q1 (<0.7222), Q2 (0.7222–1.0674), Q3 (1.0674–1.5632), and Q4 (≥1.5632). As SIRI increased, the all-cause mortality and cardiovascular mortality rates of OSA patients also increased. There were statistically significant differences among different SIRI groups in age, sex, race, PIR, education level, smoking status, drinking status, BMI, hypertension, diabetes, history of stroke, history of coronary heart disease, total cholesterol level, and eGFR (*p* < 0.05), see Table 1.

**Table 1:** Characteristics of study participants (*N* = 9,992).

Characteristic	Total participants	Q1	Q2	Q3	Q4	*p*-value
	(*N* = 9,992)	(*N* = 2,492)	(*N* = 2,504)	(*N* = 2,498)	(*N* = 2,498)	
Age, year, median (IQR)	52 (38, 64)	50 (37, 61)	50 (37, 63)	51 (39, 65)	55 (39, 69)	<0.001
Age group, *n* (%)						<0.001
(20, 40)	2,804 (28.1)	753 (30.2)	735 (29.4)	672 (26.9)	644 (25.8)	
(40, 60)	3,666 (36.7)	990 (39.2)	969 (38.7)	934 (37.4)	773 (30.9)	
≥60	3,522 (35.2)	749 (30.1)	800 (31.9)	892 (35.7)	1,081 (43.3)	
Sex, *n* (%)						<0.001
Male	5,374 (53.8)	1,164 (46.7)	1,285 (51.3)	1,436 (57.5)	1,489 (59.6)	
Female	4,618 (46.2)	1,328 (53.3)	1,219 (48.7)	1,062 (42.5)	1,009 (40.4)	
Educational level, *n* (%)						0.007
<High school graduate	5,054 (50.6)	1,206 (48.4)	1,238 (49.5)	1,292 (51.8)	1,318 (52.8)	
High school or above	4,932 (49.4)	1,285 (51.6)	1,265 (50.5)	1,203 (48.2)	1,179 (47.2)	
Race/Ethnicity, *n* (%)						<0.001
White	4,076 (40.8)	592 (23.8)	982 (39.2)	1,168 (46.8)	1,334 (53.4)	
Non-White	5,916 (59.2)	1,900 (76.2)	1,522 (60.8)	1,330 (53.2)	1,164 (46.6)	
PIR, *n* (%)						<0.001
<1.3	2,637 (28.8)	688 (30.1)	605 (26.4)	661 (29.1)	683 (29.6)	
(1.3, 3.5)	3,676 (40.2)	888 (38.9)	911 (39.8)	897 (39.5)	980 (42.5)	
≥3.5	2,835 (31.0)	707 (31.0)	773 (33.8)	714 (31.4)	641 (27.8)	
Marital status, *n* (%)						0.015
Married/Living with partner	6,607 (66.1)	1,322 (45.4)	1,683 (67.2)	1,675 (67.1)	1,586 (63.5)	
Widowed/Divorced/Separated/Never married	3,382 (33.9)	1,587 (54.6)	820 (32.8)	822 (32.9)	911 (36.5)	
Smoker, *n* (%)	4,899 (49.1)	1,033 (41.5)	1,157 (46.2)	1,259 (50.4)	1,450 (58.1)	<0.001
Alcohol drinking, *n* (%)	7,158 (76.7)	1,733 (74.5)	1,767 (75.5)	1,787 (77.1)	1,871 (79.8)	<0.001
BMI category, *n* (%)						<0.001
Normal	1,950 (19.8)	523 (21.2)	495 (20.0)	465 (18.9)	467 (19.1)	
Overweight	3,224 (32.8)	879 (35.6)	830 (35.5)	781 (31.8)	734 (30.0)	
Obese	4,666 (47.4)	1,066 (43.2)	1,149 (46.4)	1,208 (49.2)	1,243 (50.9)	
Hypertension, *n* (%)	4,070 (40.8)	929 (37.3)	932 (37.3)	1,035 (41.5)	1,174 (47.1)	<0.001
Diabetes mellitus, *n* (%)	1,599 (16.0)	354 (14.2)	341 (13.6)	410 (16.4)	494 (19.8)	<0.001
Stroke, *n* (%)	465 (4.7)	97 (3.9)	73 (2.9)	114 (4.6)	181 (7.3)	<0.001
Coronary heart disease, *n* (%)	520 (5.2)	91 (3.7)	91 (3.6)	120 (4.8)	218 (8.7)	<0.001
eGFR	95.5 (78.8, 109.0)	96.4 (80.9, 109.1)	97.0 (81.1, 109.3)	95.4 (79.2, 109.3)	92.6 (73.2, 107.8)	<0.001
Cholesterol	192.0 (166.0, 222.0)	196.0 (169.0, 225.0)	194.0 (167.8, 222.0)	193.0 (167.0, 224.0)	186.0 (161.0, 217.0)	<0.001
All-cause mortality	1,040 (10.4)	167 (6.7)	190 (7.6)	272 (10.9)	411 (16.5)	<0.001
Cardiovascular mortality	233 (2.3)	37 (1.5)	30 (1.2)	59 (2.4)	107 (4.3)	<0.001

IQR, interquartile range; eGFR, estimated glomerular filtration rate; BMI, body mass index; PIR, family poverty income ratio.

### 3.2 SIRI and all-cause mortality and cardiovascular mortality in individuals with OSA symptoms

For all-cause mortality, in the fully adjusted model (Model C), when SIRI was treated as a continuous variable, it was significantly positively associated with all-cause mortality risk in the OSA population (HR = 1.20, 95% CI: 1.12–1.29); compared with the Q1 group, all-cause mortality risk was significantly higher in the Q4 group (HR = 1.51, 95% CI: 1.16–1.97), with *p* for trend <0.001 (see Table 2).

**Table 2:** Associations between SIRI and the risk of all-cause mortality^a^.

		Model A			Model B			Model C	
	HR	95% CI	*p*-value	HR^a^	95% CI	*p*-value	HR	95% CI	*p*-value
Continuous	1.45	1.37–1.53	<0.001	1.27	1.20–1.35	<0.001	1.20	1.12–1.29	<0.001
Q1	Reference			Reference			Reference		
Q2	0.93	0.73–1.20	0.597	0.82	0.63–1.07	0.146	0.91	0.69–1.19	0.488
Q3	1.72	1.39–2.12	<0.001	1.38	1.06–1.79	0.015	1.31	0.95–1.80	0.101
Q4	2.81	2.24–3.52	<0.001	1.82	1.42–2.34	<0.001	1.51	1.16–1.97	0.002
*p*-trend			<0.001			<0.001			<0.001

CI, confidence interval; HR, hazards ratio.

a The associations are presented as HRs (95% CI). Model A did not adjust for any covariates. Model B adjusted for age, race, sex. Model C further adjusted for education level, marital status, the ratio of family income to poverty, BMI, alcohol use, smoking, stroke, cholesterol, eGFR, hypertension, diabetes, coronary heart disease based on Model B.

For cardiovascular mortality, in Model C, continuous form of SIRI was significantly positively associated with cardiovascular mortality risk in the OSA population (HR = 1.29, 95% CI: 1.15–1.44); compared with the Q1 group, the Q4 group (HR = 2.03, 95% CI: 1.17–3.54) showed significantly higher cardiovascular mortality risk, with *p* for trend = 0.002 (see Table 3).

**Table 3:** Associations between SIRI and the risk of cardiovascular mortality^a^.

		Model A			Model B			Model C	
	HR	95% CI	*p*-value	HR	95% CI	*p*-value	HR	95% CI	*p*-value
Continuous	1.57	1.46–1.68	<0.001	1.35	1.24–1.47	<0.001	1.29	1.15–1.44	<0.001
Q1	Reference			Reference			Reference		
Q2	0.66	0.336–1.29	0.225	0.56	0.29–1.10	0.089	0.71	0.34–1.49	0.369
Q3	1.90	1.20–3.00	0.006	1.42	0.88–2.28	0.147	1.36	0.78–2.37	0.275
Q4	3.89	2.60–5.81	<0.001	2.22	1.41–3.49	<0.001	2.03	1.17–3.54	0.012
*p*-trend			<0.001			<0.001			0.002

CI, confidence interval; HR, hazards ratio.

a The associations are presented as HRs (95% CI). Model A did not adjust for any covariates. Model B adjusted for age, race, sex. Model C further adjusted for education level, marital status, the ratio of family income to poverty, BMI, alcohol use, smoking, stroke, cholesterol, eGFR, hypertension, diabetes, coronary heart disease based on Model B.

### 3.3 Subgroup analysis in NHANES dataset

For all-cause mortality, SIRI was associated with an increased risk of all-cause mortality. This association was evident in the following subgroups: individuals aged 40–59 years (HR = 1.24, 95% CI: 1.01–1.52); those aged ≥60 years (HR = 1.26, 95% CI: 1.18–1.35); those overweight (HR = 1.24, 95% CI: 1.12–1.37); and obese (HR = 1.22, 95% CI: 1.03–1.45). Moreover, regardless of smoking status, drinking status, hypertension, or diabetes status, higher SIRI levels were associated with an increased risk of all-cause mortality. Additionally, there was a significant interaction between diabetes status and SIRI in their association with all-cause mortality (*p* for interaction <0.001).

**Figure 2:**
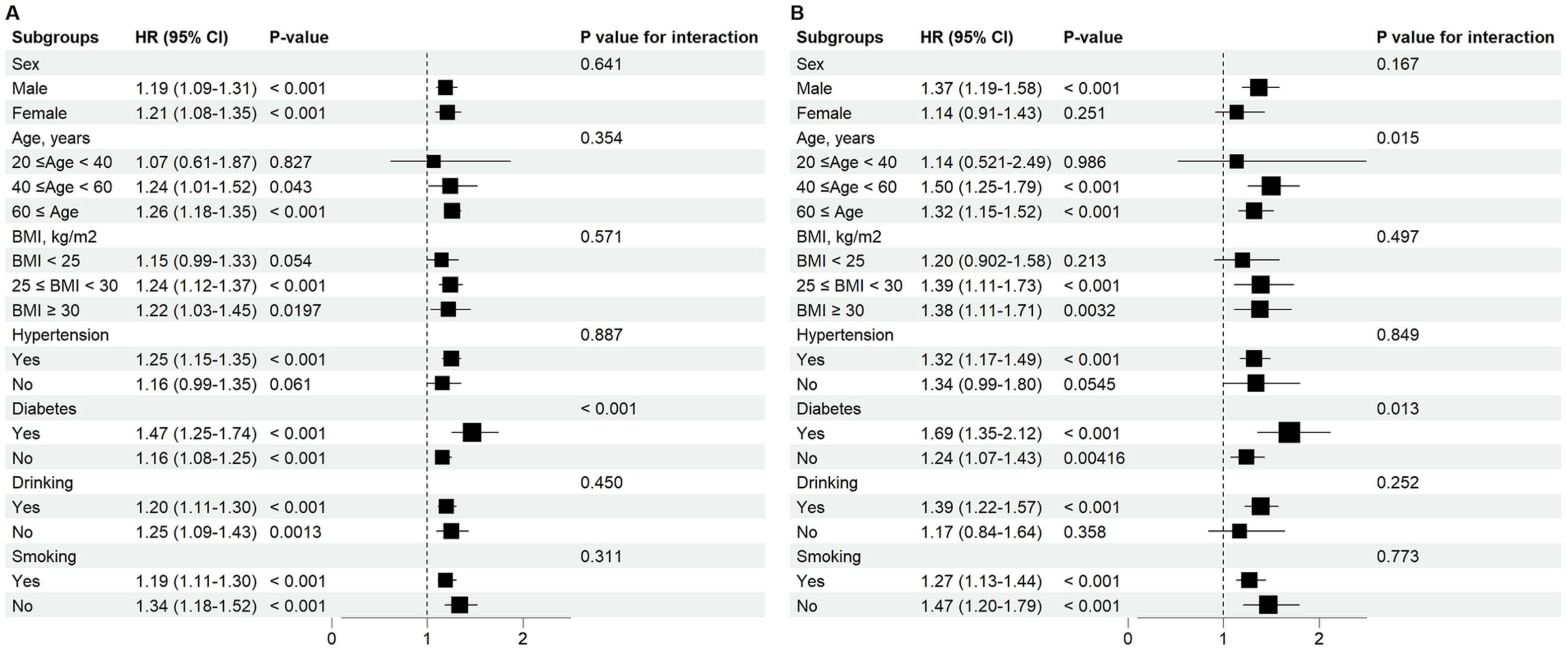
Subgroup analysis of the relationship between SIRI and all-cause/cardiovascular mortality in OSA. **(A)** All-cause mortality. **(B)** Cardiovascular mortality.

For cardiovascular mortality, the association between elevated SIRI and increased cardiovascular mortality risk was observed in the following subgroups: males (HR = 1.37, 95% CI: 1.19–1.58); those aged 40–59 years (HR = 1.50, 95% CI: 1.25–1.79); those aged ≥60 years (HR = 1.32, 95% CI: 1.15–1.52); those overweight (HR = 1.39, 95% CI: 1.11–1.73) or obese (HR = 1.38, 95% CI: 1.11–1.71); those with hypertension (HR = 1.32, 95% CI: 1.17–1.49); and those with positive drinking status (HR = 1.39, 95% CI: 1.22–1.57). This association remained significant regardless of smoking status or diabetes status. It was noteworthy that diabetes status (*p* for interaction = 0.013) and age group (*p* for interaction = 0.015) showed significant interactions with SIRI with respect to cardiovascular mortality, see Figure 2.

### 3.4 Dose–response relationship in NHANES dataset

**Figure 3:**
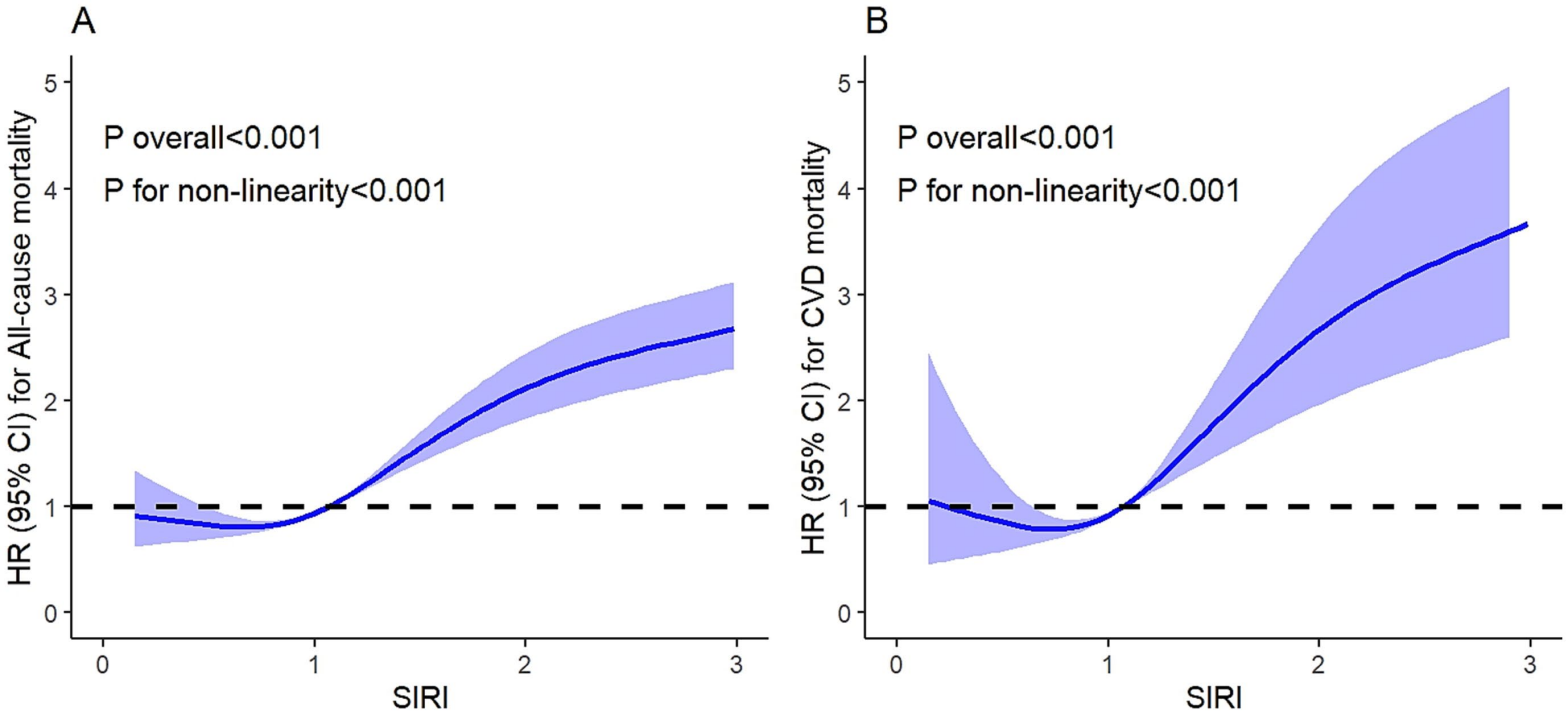
The non-linear relationship between SIRI and all-cause/cardiovascular mortality in OSA. **(A)** All-cause mortality. **(B)** Cardiovascular mortality.

As shown in Figure 3, after full adjustment, the non-linear relationship between SIRI and all-cause mortality was statistically significant (*p* for overall <0.001, *p* for non-linearity <0.001). Similarly, the non-linear relationship between SIRI and cardiovascular mortality was statistically significant (*p* for overall <0.001, *p* for non-linearity <0.001).

### 3.5 Predictive value of SIRI for all-cause mortality and cardiovascular mortality in NHANES dataset

**Figure 4:**
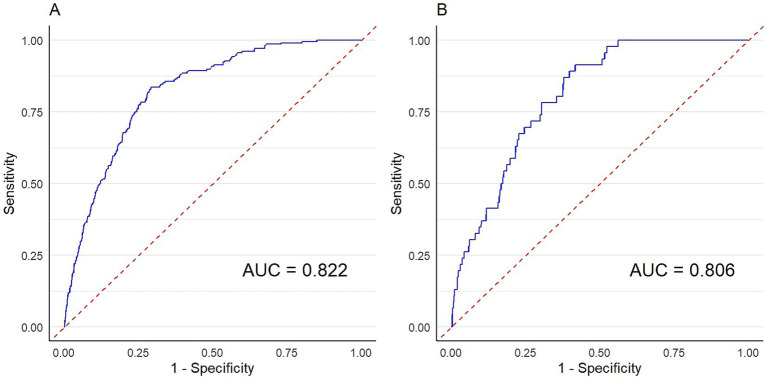
Predictive value of SIRI for all-cause/cardiovascular mortality in individuals with OSA symptoms. **(A)** All-cause mortality. **(B)** Cardiovascular mortality.

In Figure 4, for the prediction of all-cause mortality, SIRI had significant predictive value for all-cause mortality in the OSA population (AUC = 0.822); for the prediction of cardiovascular mortality, SIRI could also statistically effectively predict cardiovascular mortality risk in the OSA population (AUC = 0.806). Notably, the AUC for hs-CRP was 0.725 for all-cause mortality and 0.704 for cardiovascular mortality, both of which were lower than those of SIRI (Supplementary Figure 2). DeLong’s test confirmed that the differences between SIRI and hs-CRP were statistically significant for both outcomes (both *p* < 0.001), indicating that SIRI exhibited superior predictive capacity for mortality risk in individuals with OSA symptoms. In addition, we compared the predictive performance of SIRI with that of body mass index (BMI), a simple anthropometric measure closely linked to systemic inflammation. The AUC for BMI was 0.660 for all-cause mortality and 0.678 for cardiovascular mortality, both substantially lower than those of SIRI. DeLong’s test confirmed that the differences between SIRI and BMI were statistically significant for both outcomes (both *p* < 0.001), further supporting the superior predictive capacity of SIRI (Supplementary Figure 3).

### 3.6 External hospital data validation

This study ultimately included 994 patients with clinically diagnosed OSA. Among them, 589 were male (59.3%), with a median age of 51 years (IQR, 41–63 years), and 331 participants (33.3%) were aged 60 years or older. During a median follow-up period of 4.43 years (IQR, 2.23–6.1), 69 deaths from all causes were recorded (all-cause mortality rate: 6.9%), including 16 deaths attributable to cardiovascular diseases (cardiovascular mortality rate: 1.6%).

The baseline characteristics of the study population are summarized as follows. Regarding demographic characteristics, the majority of participants were of Han ethnicity (95.4%), and 56.1% had attained a high school education or above. In terms of marital status, 66.1% were married or living with a partner. Concerning lifestyle factors, 51.6% of participants were smokers, and 78.4% reported alcohol consumption. Based on BMI classification, 50.5% were classified as obese, 34.3% as overweight, and only 15.2% had a normal BMI. Regarding comorbidities, 36.9% had hypertension, 15.9% had diabetes mellitus, 4.7% had a history of stroke, and 4.0% had a history of coronary heart disease. The median eGFR was 96.1 mL/min/1.73 m^2^ (IQR, 84.3–109.0), and the median total cholesterol level was 194.0 mg/dL (IQR, 171.0–226.0) as detailed in Supplementary Table 3.

The associations between SIRI and the risks of all-cause and cardiovascular mortality were evaluated using Cox proportional hazards models, with progressive adjustment for potential confounders (Supplementary Tables 4, 5).

For all-cause mortality, when SIRI was modeled as a continuous variable, a significant positive association was observed in the unadjusted Model A (HR = 1.37, 95% CI: 1.13–1.65, *p* = 0.001). This association remained consistent after adjusting for age, race, and sex in Model B (HR = 1.35, 95% CI: 1.12–1.64, *p* = 0.002) and persisted in the fully adjusted Model C, which further accounted for education level, marital status, economic status, BMI, alcohol consumption, smoking status, total cholesterol, eGFR, hypertension, diabetes, and coronary heart disease (HR = 1.34, 95% CI: 1.05–1.70, *p* = 0.019). In quartile-based analyses using the lowest quartile (Q1) as the reference, participants in the highest quartile (Q4) demonstrated a significantly elevated risk of all-cause mortality across all models. Specifically, compared with Q1, the Q4 group showed HRs of 2.03 (95% CI: 1.03–3.98, *p* = 0.041) in Model A, 2.00 (95% CI, 1.02–3.94, *p* = 0.044) in Model B, and 2.08 (95% CI, 1.04–4.13, *p* = 0.038) in Model C. Although the intermediate quartiles (Q2 and Q3) did not reach statistical significance, a clear dose–response pattern was evident, with significant trends across quartiles confirmed in all models (*p* for trend = 0.012 in Model A, 0.014 in Model B, and 0.013 in Model C).

For cardiovascular mortality, similar patterns were observed. In the continuous analysis, SIRI was significantly associated with an increased risk of cardiovascular mortality in the fully adjusted Model C (HR = 1.37, 95% CI: 1.02–1.89, *p* = 0.041). In quartile-based analyses, compared with the lowest quartile (Q1), participants in the highest quartile (Q4) exhibited a substantially elevated risk of cardiovascular mortality in Model C (HR = 1.15, 95% CI: 1.34–3.87, *p* = 0.025). A significant dose–response trend across quartiles was confirmed in Model C (*p* for trend = 0.015). These findings collectively indicate that elevated SIRI levels are independently and positively associated with increased risks of both all-cause and cardiovascular mortality, with consistent results across continuous and categorical analyses, comprehensive covariate adjustment.

As shown in Supplementary Figure 4, the dose–response relationships between SIRI and the risks of all-cause and cardiovascular mortality were evaluated using restricted cubic spline regression within the fully adjusted model. After comprehensive adjustment for potential confounders, a statistically significant non-linear association was observed between SIRI levels and all-cause mortality (*p* for overall association <0.001; *p* for non-linearity <0.001). Similarly, the association between SIRI and cardiovascular mortality also exhibited a significant non-linear pattern (*p* for overall association <0.001; *p* for non-linearity <0.001). These findings indicate that the relationship between SIRI and mortality outcomes is non-linear, characterized by progressively increasing hazard ratios at higher SIRI levels.

As illustrated in Supplementary Figure 5, the predictive performance of SIRI for mortality outcomes was evaluated using ROC curve analysis in the OSA population. For the prediction of all-cause mortality (Panel A), SIRI demonstrated good predictive value, with an area under the curve (AUC) of 0.780. For cardiovascular mortality (Panel B), SIRI also exhibited effective discriminative ability, yielding an AUC of 0.866. These findings indicate that SIRI possesses statistically significant predictive capacity for both all-cause and cardiovascular mortality risk in individuals with OSA.

## 4 Discussion

Previous studies have consistently documented associations between SIRI and mortality across diverse patient cohorts. For instance, a recent investigation revealed that elevated SIRI levels are independently linked to higher risks of all-cause and cardiovascular mortality in hypertensive patients, underscoring the prognostic potential of this inflammatory index in this population (26). Similarly, a large-scale prospective study examined the combined prognostic value of inflammatory markers, including SIRI, for all-cause and cardiovascular mortality across the spectrum of cardiovascular-kidney-metabolic syndrome, demonstrating that increased SIRI reliably predicts adverse survival outcomes (27). Further evidence from diabetic and prediabetic cohorts suggests that oxidative stress and inflammation mediate the adverse impacts of cadmium exposure on mortality, with SIRI serving as a robust surrogate marker for inflammation-related risk (28). Collectively, these findings establish SIRI as a promising biomarker for mortality prediction in various chronic inflammatory diseases.

However, no study to date has specifically focused on OSA—a clinical entity characterized by chronic intermittent hypoxia, sleep fragmentation, and a unique systemic inflammatory profile that may fundamentally modulate the relationship between SIRI and long-term clinical outcomes. Furthermore, prior research has failed to investigate the potential non-linear (threshold) effect of SIRI on mortality in OSA patients, nor has it validated findings in an independent, external cohort of clinically diagnosed individuals. To our knowledge, the present study is the first to address these gaps. Specifically, we investigated the association between SIRI and both all-cause and cardiovascular mortality in a large, nationally representative cohort of adults with OSA symptoms (NHANES) and further validated the association in an independent hospital cohort of 994 Chinese patients with polysomnography-confirmed OSA from the Central Hospital of Wuhan. Our findings demonstrate that SIRI is an independent risk factor for all-cause and cardiovascular mortality at both the population level and in clinical settings, exhibiting a non-linear relationship with a well-defined threshold effect: mortality risk remains relatively stable below the threshold but rises sharply above it. Moreover, SIRI demonstrated good predictive value for mortality risk stratification in individuals with OSA. The external validation confirms the robustness and generalizability of the observed associations across different populations and clinical settings. Consequently, while existing literature validates SIRI as a mortality predictor in other chronic conditions, our work advances this knowledge by establishing SIRI as a reliably validated, OSA-specific prognostic biomarker, with significant implications for clinical risk stratification and timely therapeutic intervention.

This study focused on the positive association between SIRI and all-cause mortality and cardiovascular mortality in OSA. This suggests that SIRI can be one of the important indicators for evaluating the prognosis risk of OSA patients, providing new evidence for clinicians to judge the condition and make treatment decisions for OSA patients. Previous studies have shown that in patients with CKD, elevated SIRI was an independent risk factor for all-cause mortality and cardiovascular mortality, and its significance was more pronounced in the early stage of CKD (13), which indicates that SIRI is closely related to the risk of death, but our study firstly focused on the OSA population, further expanding the application scope of SIRI as a mortality risk predictor, suggesting that SIRI may play an important role in different disease contexts and has certain universal value in the prognosis assessment of chronic diseases. For comparison with studies on the osteoarthritis (OA) population, a prospective cohort study on OA patients also found that higher SIRI was significantly associated with increased all-cause mortality and cardiovascular mortality in adult OA patients in the US (29). The results of our study are consistent with this, emphasizing the association between SIRI and the risk of death in different chronic disease populations, and both are independent correlations. This further confirms the stability and effectiveness of SIRI as an inflammatory-related indicator in predicting the adverse prognosis of chronic disease patients and provides ideas and references for exploring the predictive value of SIRI in more disease fields in the future.

The repeated apneas and hypopneas during sleep in OSA patients led to intermittent hypoxia and hypercapnia, activating inflammatory responses and generating large amounts of pro-inflammatory factors (e.g., IL-6, TNF-*α*, CRP) (30, 31), these factors promote the aggregation and activation of inflammatory cells, triggering systemic inflammation. SIRI combined the counts of neutrophils, monocytes, and lymphocytes, and its increase directly reflected a strong inflammatory state, causing continuous damage to tissues and organs and increasing the risk of cardiovascular diseases and mortality risk (32). At the same time, the hypoxia-reoxygenation process generated large amounts of reactive oxygen species (ROS) (33) which causes oxidative stress damage. Oxidative stress not only aggravates inflammatory responses but also leads to vascular endothelial dysfunction and lipid peroxidation, promoting the development of atherosclerosis and increasing the risk of cardiovascular events (34, 35). The changes in the number and proportion of inflammatory cells in response to oxidative stress also affected SIRI, making it an indirect indicator of oxidative stress damage and oxidative-antioxidant imbalance, and is intrinsically associated with cardiovascular mortality risk. Repeated hypoxia also stimulated excessive activation of the sympathetic nervous system, releasing vasoactive substances (e.g., catecholamines), causing vasoconstriction, increased blood pressure, and damaging vascular endothelial function (e.g., reduced nitric oxide release) (36, 37). Vascular endothelial dysfunction is a crucial link in the occurrence and development of atherosclerosis and cardiovascular diseases. Inflammatory cells (especially neutrophils and monocytes) played a significant role in the adhesion and migration during this process, accelerating the progression of atherosclerosis (38), this can also be reflected by changes in SIRI. Moreover, long-term OSA often causes metabolic disorders (insulin resistance, hyperglycemia, dyslipidemia) (39–41). These abnormalities exacerbate inflammation and oxidative stress, forming a vicious cycle and increasing cardiovascular risks (32). Inflammatory factors themselves also regulate metabolism (such as TNF-*α* aggravating insulin resistance) (42). Therefore, an increase in SIRI may also reflect an inflammatory state related to metabolic disorders, and has a complex interaction with increased mortality, jointly influencing the prognosis of OSA patients.

Subgroup analyses revealed significant interactions only for diabetes status (for both all-cause and cardiovascular mortality) and for age group (for cardiovascular mortality), indicating that these factors may modify the prognostic value of SIRI in patients with OSA. This was similar to the results of several meta-analyses (43, 44), especially in subgroups such as the elderly and those with diabetes, the predictive value of SIRI for mortality risk was more prominent. This suggests that in clinical practice, targeted monitoring (such as focusing on the SIRI level of middle-aged and elderly male obese OSA patients) can be carried out based on this. The core mechanism of this phenomenon is that there is a “three-level cascade amplification effect of inflammation-hypoxia-metabolism” in the relevant population: IL-6/TNF-*α* (45) released by visceral fat in obese individuals, together with the NF-κB pathway activated by hypoxia caused by OSA, jointly trigger the systemic activation of neutrophils/macrophages, forming a chronic inflammatory storm (46). At the same time, the ROS burst caused by hypoxia-reoxygenation and vascular endothelial dysfunction accelerates atherosclerosis; metabolic disorders further activate inflammation through insulin resistance and lipotoxicity (35), and SIRI could directly capture the proliferation and migration of inflammatory cells.

As a composite inflammatory indicator, the nonlinear relationship discovered in this study indicates that SIRI and mortality are not simply linear, and there is a “threshold effect.” When the level is below the threshold, the risk is relatively stable, and when it exceeds the threshold, the risk significantly increases. Furthermore, validation using the hospital cohort corroborated the findings from the NHANES, indicating that SIRI may serve as a reliable prognostic biomarker for long-term outcomes in Chinese patients with OSA. This suggests that in clinical practice, the critical value of SIRI needs to be identified, and timely intervention (such as anti-inflammatory treatment, improvement of OSA condition, etc.) should be carried out for patients with excessively high SIRI levels to reduce the risk of death, at the same time, the nonlinear characteristic also reminds us that curve fitting should be considered in the research to avoid bias caused by linear assumptions, providing a more reliable basis for the precise prognosis assessment and treatment decision-making for OSA patients. In addition to systemic inflammatory indices, emerging evidence highlights the prognostic value of imaging-based cardiovascular risk markers in patients with OSA. Myocardial deformation parameters derived from speckle tracking echocardiography, such as global longitudinal strain (GLS), have been shown to detect subclinical cardiac dysfunction and predict adverse outcomes even in the absence of overt reductions in left ventricular ejection fraction (47). Notably, these strain-derived parameters may also correlate with specific anthropometric and metabolic phenotypes commonly observed in OSA populations, particularly android obesity, which is closely linked to both inflammatory activation and cardiovascular risk. Integrating such imaging markers with readily available inflammatory indices like SIRI may offer a complementary approach to refine risk stratification and guide personalized management in patients with OSA. Future studies are warranted to explore the combined utility of these biomarkers in large-scale, prospective cohorts.

Key strengths of this study include the use of a large, nationally representative cohort with comprehensive covariate adjustment, the application of rigorous statistical methods accounting for complex survey design, and the inclusion of an independent external validation cohort of clinically diagnosed OSA patients, which strengthens the generalizability and robustness of our findings. Several limitations of this study should be acknowledged. First, the diagnosis of OSA was based solely on self-reported questionnaire responses rather than objective polysomnographic data, which may introduce recall bias and misclassification. Second, as a cross-sectional analysis of baseline data, this study lacks longitudinal measurements of SIRI, preventing the assessment of its dynamic changes over time and their association with mortality. Third, despite adjusting for numerous potential confounders, residual confounding due to unmeasured factors (e.g., detailed medication use, severity of OSA, and treatment adherence) cannot be entirely excluded.

## 5 Conclusion

Our findings suggest that SIRI serves as a significant and independent predictor of both all-cause and cardiovascular mortality in patients with OSA, revealing a non-linear relationship between SIRI levels and mortality risk. Therefore, incorporating SIRI into routine clinical or public health assessment may improve risk stratification and prognostication in this population. To enhance clinical practice, we recommend that healthcare providers consider monitoring SIRI as part of the standard evaluation for OSA patients. Furthermore, future studies should explore whether interventions targeting reductions in SIRI—such as anti-inflammatory strategies or optimized OSA management—can improve long-term survival and quality of life. Implementing these measures could facilitate early identification of high-risk individuals and inform more personalized treatment approaches, ultimately improving clinical outcomes in OSA.

## Data Availability

The raw data supporting the conclusions of this article will be made available by the authors, without undue reservation.
