# Supplementary material for: Systemic inflammatory response index is associated with increased all-cause and cardiovascular mortality in obstructive sleep apnea: evidence from NHANES and validation with an external hospital-based dataset
Source: Front Med (Lausanne). 2026 May 1;13:1823845. doi: 10.3389/fmed.2026.1823845 (PMC13176312; doi:10.3389/fmed.2026.1823845)
Supplement: Supplementary file 1 [file Data_Sheet_1.docx]

| Supplementary Table 1 The results of the multicollinearity test on the NHANES data. | | |
| --- | --- | --- |
| Variables | VIF | |
|  | All-cause mortality | CVD mortality |
| SIRI | 1.073 | 1.073 |
| Age | 2.097 | 2.097 |
| Sex | 1.15 | 1.15 |
| Race | 1.126 | 1.126 |
| Educational level | 1.186 | 1.186 |
| Marital status | 1.077 | 1.077 |
| PIR | 1.258 | 1.258 |
| Smoker | 1.297 | 1.297 |
| Alcohol drinking | 1.137 | 1.137 |
| BMI category | 1.108 | 1.108 |
| Hypertension | 1.294 | 1.294 |
| Diabetes mellitus | 1.187 | 1.187 |
| Coronary heart disease | 1.376 | 1.376 |
| Stroke | 1.08 | 1.08 |
| cholesterol | 1.054 | 1.054 |
| eGFR | 1.85 | 1.85 |

| Supplementary Table 2 The results of multicollinearity tests on the validation data. | | |
| --- | --- | --- |
| Variables | VIF | |
|  | All-cause mortality | CVD mortality |
| SIRI | 1.073 | 1.073 |
| Age | 2.097 | 2.097 |
| Sex | 1.15 | 1.15 |
| Race | 1.126 | 1.126 |
| Educational level | 1.186 | 1.186 |
| Marital status | 1.077 | 1.077 |
| PIR | 1.258 | 1.258 |
| Smoker | 1.297 | 1.297 |
| Alcohol drinking | 1.137 | 1.137 |
| BMI category | 1.108 | 1.108 |
| Hypertension | 1.294 | 1.294 |
| Diabetes mellitus | 1.187 | 1.187 |
| Coronary heart disease | 1.376 | 1.376 |
| Stroke | 1.08 | 1.08 |
| cholesterol | 1.054 | 1.054 |
| eGFR | 1.85 | 1.85 |


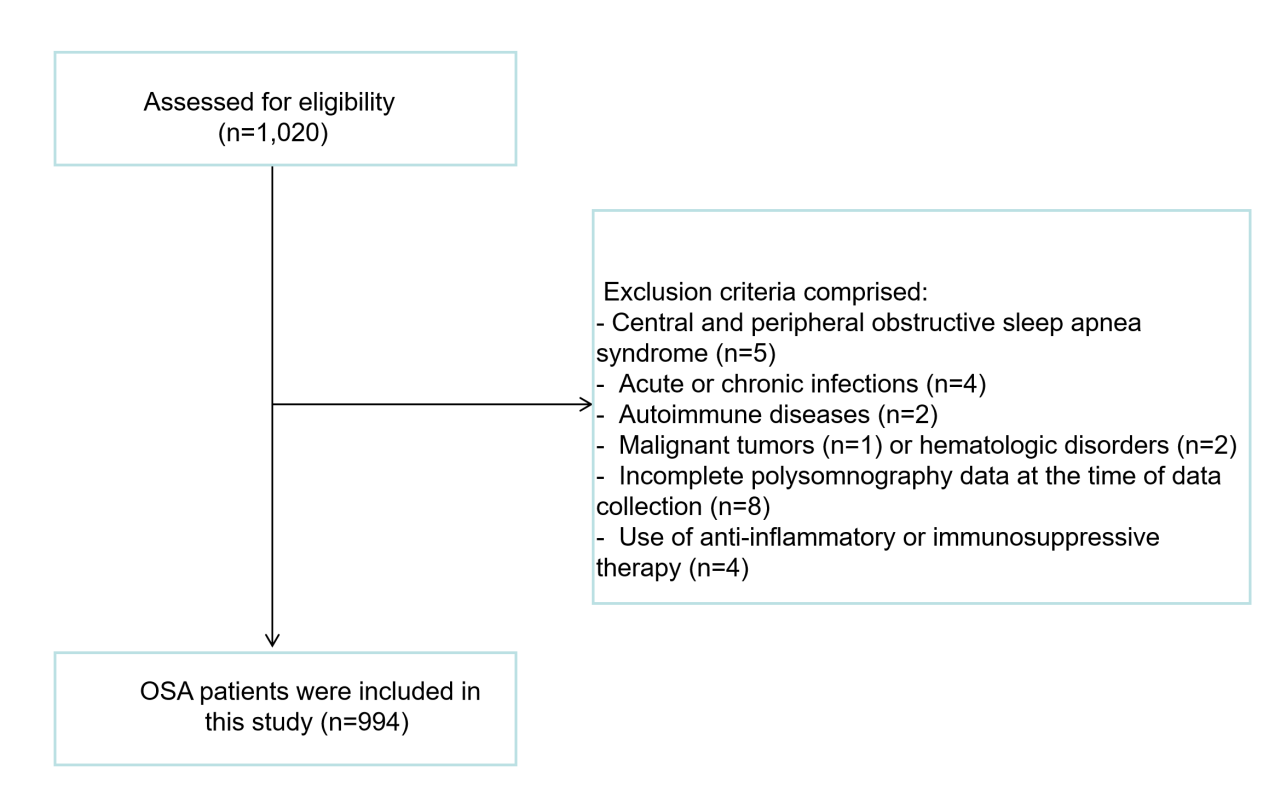


Supplementary Fig. 1. Flowchart of patient inclusion and exclusion.


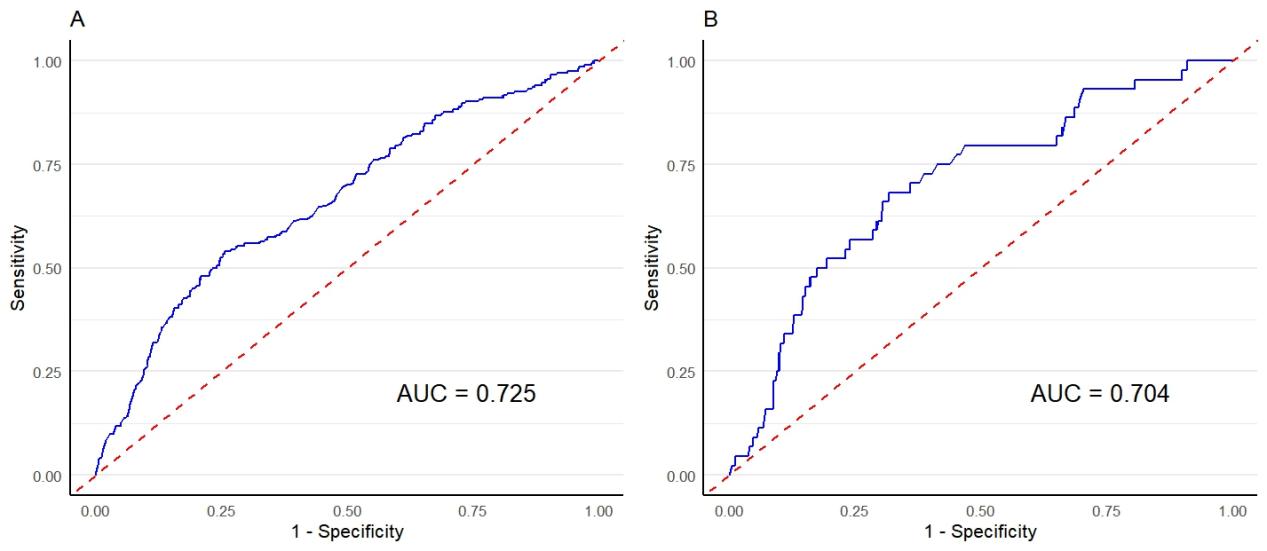


Supplementary Fig. 2. Predictive value of hs-CRP for all-cause/cardiovascular mortality in individuals with OSA symptoms. (A) all-cause mortality; (B) cardiovascular mortality.


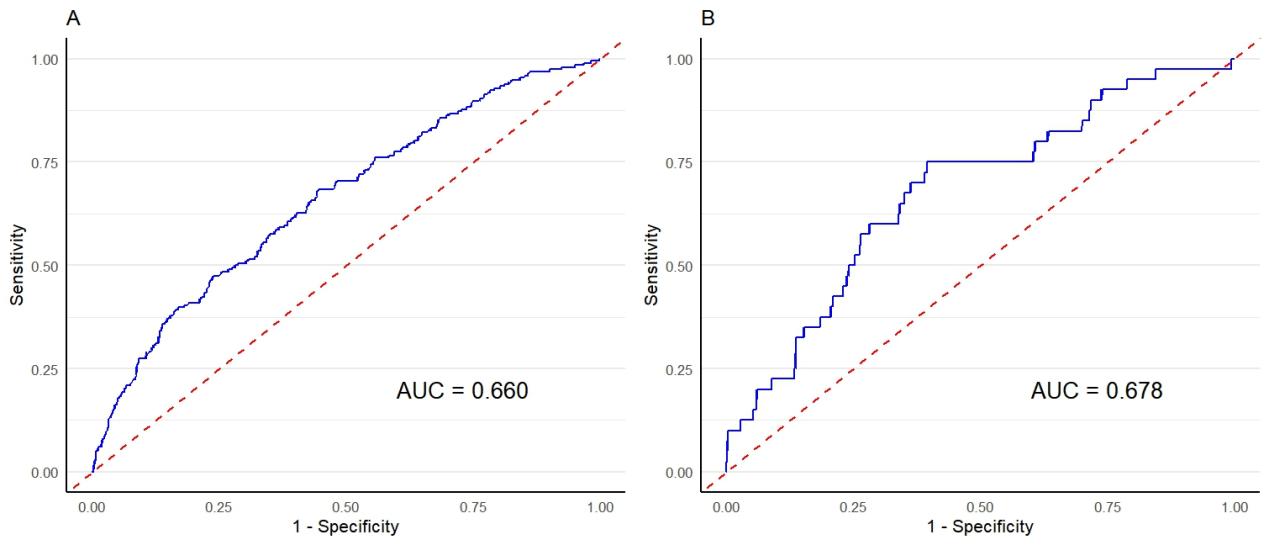


Supplementary Fig. 3. Predictive value of BMI for all-cause/cardiovascular mortality in individuals with OSA symptoms. (A) all-cause mortality; (B) cardiovascular mortality.

| Supplementary Table 3. Characteristics of the validation group. | |
| --- | --- |
| Characteristic | Total participants |
|  | (N=994) |
| **Age, y, median (IQR)** | 51 (41,63) |
| **Age group, n(%)** |  |
| [20,40) | 213 (21.4) |
| [40,60) | 450 (45.3) |
| ≥60 3 | 331 (33.3) |
| **Sex, n (%)** |  |
| Male | 589 (59.3) |
| Female | 405 (40.7) |
| **Educational level, n (%)** |  |
| <High school graduate | 436(43.9) |
| High school or above | 558(56.1) |
| **Ethnicity, n (%)** |  |
| Han | 948 (95.4) |
| Minority | 46 (4.6) |
| **Economic status, n (%)** |  |
| Poor | 2637 (28.8) |
| Ordinary | 3676 (40.2) |
| Rich | 2835 (31.0) |
| **Marital status, n (%)** |  |
| Married/Living with partner | 6607 (66.1) |
| Widowed/Divorced/Separated/Never married | 3382 (33.9) |
| **Smoker, n (%)** | 512 (51.6) |
| **Alcohol drinking, n (%)** | 748 (78.4) |
| **BMI category, n (%)** |  |
| Normal | 151 (15.2) |
| Overweight | 341 (34.3) |
| Obese | 502 (50.5) |
| **Hypertension, n (%)** | 366 (36.9) |
| **Diabetes mellitus, n (%)** | 158 (15.9) |
| **Stroke, n (%)** | 465 (4.7) |
| **Coronary heart disease, n (%)** | 40 (4.0) |
| **eGFR, median (IQR)** | 96.1 (84.3,109.0) |
| **Cholesterol, median (IQR)** | 194.0 (171.0–226.0) |
| **All-cause mortality** | 69(6.9) |
| **Cardiovascular mortality** | 16 (1.6) |

| Supplementary Table 4. Associations between SIRI and the risk of all-cause mortality in the hospital dataset ^a^. | | | | | | | | | |  |
| --- | --- | --- | --- | --- | --- | --- | --- | --- | --- | --- |
|  |  | Model A |  |  | Model B |  |  | Model C |  |  |
|  | HR | 95% CI | p-Value | HR | 95% CI | p-Value | HR | 95% CI | p-Value |  |
| Continuous | 1.37 | 1.13-1.65 | 0.001 | 1.35 | 1.12-1.64 | 0.002 | 1.34 | 1.05-1.70 | 0.019 |  |
| Q1 | Reference |  |  | Reference |  |  | Reference |  |  |  |
| Q2 | 0.87 | 0.40-1.92 | 0.743 | 0.88 | 0.40-1.94 | 0.759 | 0.95 | 0.43-2.07 | 0.888 |  |
| Q3 | 1.51 | 0.75-3.04 | 0.246 | 1.50 | 0.74-3.01 | 0.258 | 1.59 | 0.79-3.22 | 0.101 |  |
| Q4 | 2.03 | 1.03-3.98 | 0.041 | 2.00 | 1.02-3.94 | 0.044 | 2.08 | 1.04-4.13 | 0.038 |  |
| p-trend |  |  | 0.012 |  |  | 0.014 |  |  | 0.013 |  |
| Abbreviations: CI, confidence interval; HR, hazards ratio. ^a^ The associations are presented as HRs (95% CI). Model A did not adjust for any covariates. Model B adjusted for age, race, sex. Model C further adjusted for education level, marital status, economic status, BMI, alcohol use, smoking—cigarette use, stroke, cholesterol, eGFR, hypertension, diabetes, coronary heart disease based on Model B. | | | | | | | | | |  |
|  |  |  |  |  |  |  |  |  |  |  |
|  |  |  |  |  |  |  |  |  |  |  |

| Supplementary Table 5. Associations between SIRI and the risk of CVD mortality in the hospital dataset ^a^. | | | | | | | | | |  |
| --- | --- | --- | --- | --- | --- | --- | --- | --- | --- | --- |
|  |  | Model A |  |  | Model B |  |  | Model C |  |  |
|  | HR | 95% CI | p-Value | HR | 95% CI | p-Value | HR | 95% CI | p-Value |  |
| Continuous | 1.46 | 1.07-1.99 | 0.017 | 1.41 | 1.02-1.94 | 0.037 | 1.37 | 1.02-1.89 | 0.041 |  |
| Q1 | Reference |  |  | Reference |  |  | Reference |  |  |  |
| Q2 | 1.19 | 0.92-1.63 | 0.130 | 1.20 | 0.72-1.68 | 0.137 | 1.20 | 0.42-1.75 | 0.147 |  |
| Q3 | 1.59 | 0.84-2.48 | 0.474 | 1.57 | 0.94-2.40 | 0.445 | 1.56 | 0.83-2.38 | 0.429 |  |
| Q4 | 1.54 | 1.11-4.86 | 0.001 | 1.48 | 1.18-4.69 | 0.009 | 1.15 | 1.34-3.87 | 0.025 |  |
| p-trend |  |  | 0.001 |  |  | 0.003 |  |  | 0.015 |  |
| Abbreviations: CI, confidence interval; HR, hazards ratio. ^a^ The associations are presented as HRs (95% CI). Model A did not adjust for any covariates. Model B adjusted for age, race, sex. Model C further adjusted for education level, marital status, economic status, BMI, alcohol use, smoking—cigarette use, stroke, cholesterol, eGFR, hypertension, diabetes, coronary heart disease based on Model B. | | | | | | | | | |  |
|  |  |  |  |  |  |  |  |  |  |  |
|  |  |  |  |  |  |  |  |  |  |  |


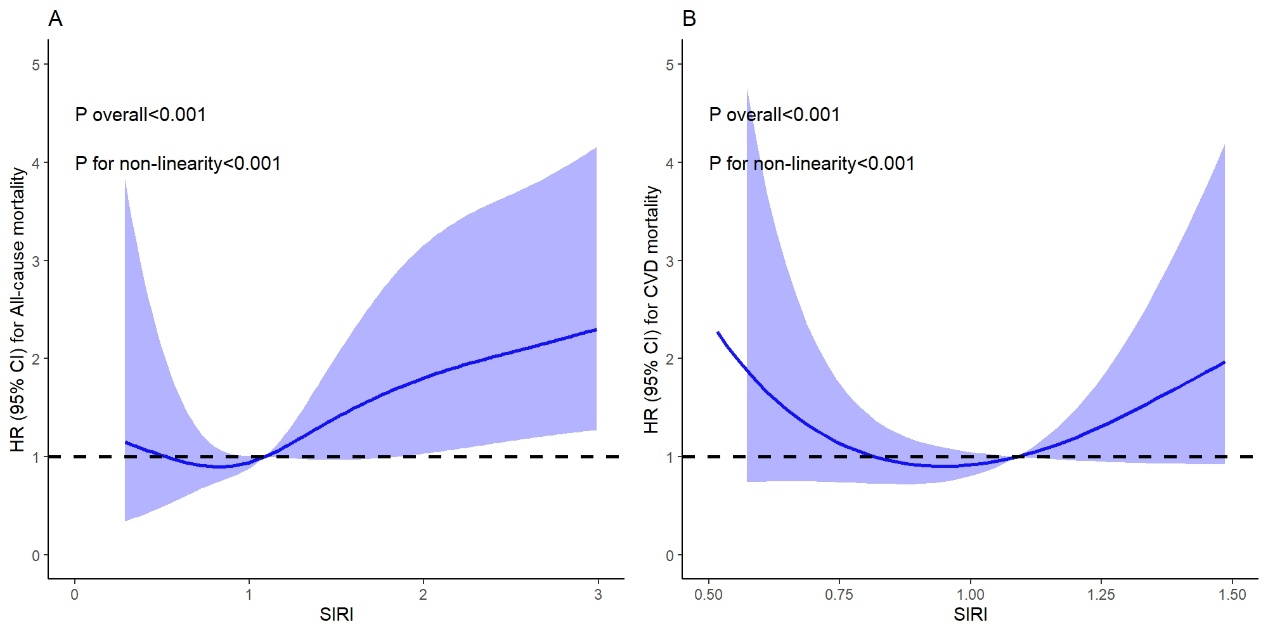


Supplementary Fig. 4. The non-linear relationship between SIRI and all-cause/cardiovascular mortality in OSA from China. (A) all-cause mortality; (B) cardiovascular mortality.


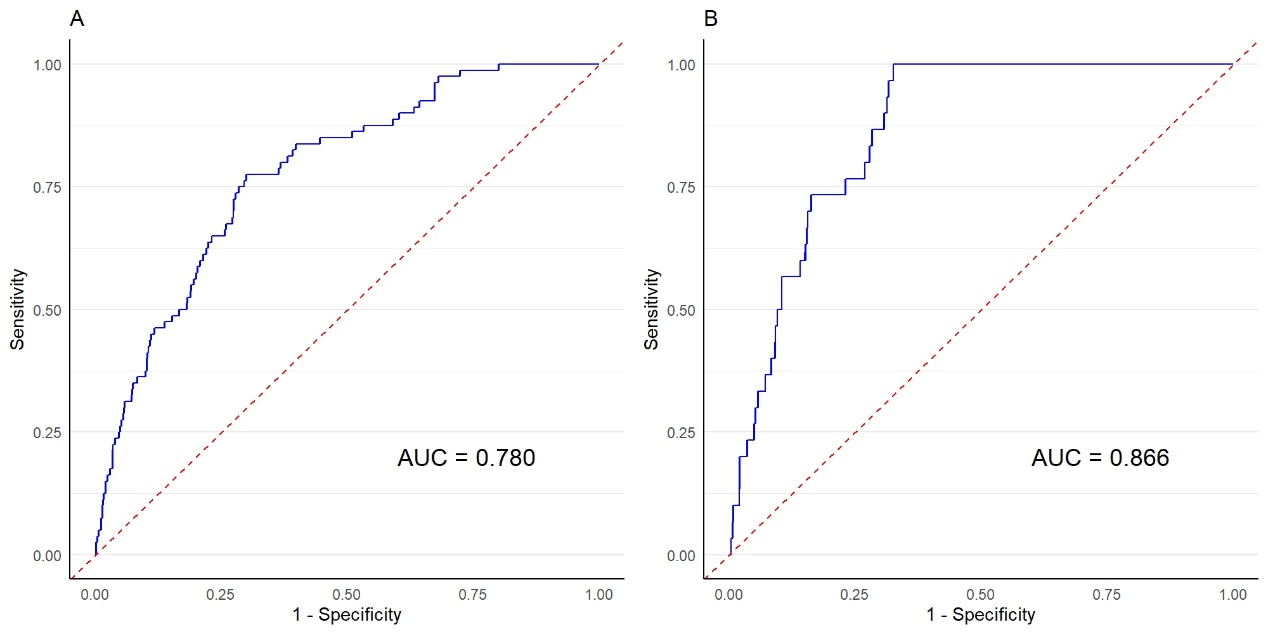


Supplementary Fig. 5. Predictive value of SIRI for all-cause/cardiovascular mortality in individuals with OSA from China. (A) all-cause mortality; (B) cardiovascular mortality.
